# Development and Validation of Prognostic Models for Treatment Response of Patients with B-Cell Lymphoma: Standard Statistical and Machine-Learning Approaches

**DOI:** 10.3390/jcm14207445

**Published:** 2025-10-21

**Authors:** Adugnaw Zeleke Alem, Itismita Mohanty, Nalini Pati, Cameron Wellard, Eliza Chung, Eliza A. Hawkes, Zoe K. McQuilten, Erica M. Wood, Stephen Opat, Theophile Niyonsenga

**Affiliations:** 1 Health Research Institute, University of Canberra, Canberra 2617, Australia; 2 Department of Epidemiology and Biostatistics, Institute of Public Health, College of Medicine and Health Sciences, University of Gondar, Gondar 196, Ethiopia; 3 Department of Haematology, The Canberra Hospital, Canberra 2605, Australia; 4 ANU Medical School, Canberra 2605, Australia; 5 School of Public Health and Preventive Medicine, Monash University, Melbourne 3004, Australia; 6 Olivia Newton-John Cancer Research Institute at Austin Health, Melbourne 3084, Australia; 7 Alfred Health, Melbourne 3004, Australia; 8 Monash Haematology, Monash Health, Melbourne 3168, Australia; 9 Department of Medicine, School of Clinical Sciences at Monash Health, Monash University, Melbourne 3004, Australia

**Keywords:** lymphoma, treatment response, machine learning, inflammatory-nutritional indicators, nomogram

## Abstract

**Background:** Achieving a complete response after therapy is an important predictor of long-term survival in lymphoma patients. However, previous predictive models have primarily focused on overall survival (OS) and progression-free survival (PFS), often overlooking treatment response. Predicting the likelihood of complete response before initiating therapy can provide more immediate and actionable insights. Thus, this study aims to develop and validate predictive models for treatment response to first-line therapy in patients with B-cell lymphomas. **Methods:** The study used 2763 patients from the Lymphoma and Related Diseases Registry (LaRDR). The data were randomly divided into training (n = 2221, 80%) and validation (n = 553, 20%) cohorts. Seven algorithms: logistic regression, K-nearest neighbor, support vector machine, random forest, Naïve Bayes, gradient boosting machine, and extreme gradient boosting were evaluated. Model performance was assessed using discrimination and classification metrics. Additionally, model calibration and clinical utility were evaluated using the Brier score and decision curve analysis, respectively. **Results:** All models demonstrated comparable performance in the validation cohort, with area under the curve (AUC) values ranging from 0.69 to 0.70. A nomogram incorporating the six variables, including stage, lactate dehydrogenase, performance status, BCL2 expression, anemia, and systemic immune-inflammation index, achieved an AUC of 0.70 (95% CI: 0.65–0.75), outperforming the international prognostic index (IPI: AUC = 0.65), revised IPI (AUC = 0.61), and NCCN-IPI (AUC = 0.63). Decision curve analysis confirmed the nomogram’s superior net benefit over IPI-based systems. **Conclusions:** While our nomogram demonstrated improved discriminative performance and clinical utility compared to IPI-based systems, further external validation is needed before clinical integration.

## 1. Introduction

Lymphoma is the most common type of hematological cancer, with non-Hodgkin lymphoma (NHL) accounting for approximately 90% of all lymphoma subtypes [1,2]. In 2020, an estimated 5.44 million people were diagnosed with NHL cases, and approximately 260,000 deaths were attributed to NHL globally [3]. Age-specific incidence rates of NHL are estimated to vary globally, with the most pronounced increasing trends observed in Australia and New Zealand [4]. Moreover, in 2019, NHL resulted in 8,650,352 age-standardized disability-adjusted life years (DALYs) globally [4].

Over the past two decades, the prognosis of lymphoma patients has been significantly improved due to advances in diagnostic tools and targeted therapies, including immunotherapy, and cellular therapies [5,6]. Developing accurate prognostic predictions to categorize patients and inform clinical decisions is essential for enhancing patient outcomes. Currently, the International Prognostic Index (IPI), its updated versions, such as the Revised-IPI (R-IPI) and the National Comprehensive Cancer Network-IPI (NCCN-IPI), are widely used for risk stratification in diffuse large B-cell lymphoma (DLBCL) patients [7,8,9]. Moreover, the Follicular Lymphoma IPI (FLIPI) and the Mantle Cell Lymphoma IPI (M-IPI) are also valuable prognostic tools for risk stratification in follicular lymphoma (FL) and mantle cell lymphoma (MCL) [10,11]. However, these prognostic tools have primarily focused on overall survival (OS) and progression-free survival (PFS) as endpoints [8,9,12,13]. While 5-year OS and PFS are important predominating endpoints for measuring treatment efficacy [14], predicting treatment response before initiating therapy can provide more immediate and actionable insights for effective management and may facilitate the development and evaluation of novel therapies [15,16]. Recent evidence has demonstrated that early treatment response is a validated surrogate endpoint for long-term survival outcomes in lymphoma [16]. This underscores the need for prognostic models that incorporate treatment response as a primary endpoint.

Achieving a complete response (CR) after the course of therapy is an important predictor of long-term survival in lymphoma patients [16,17,18,19]. Although the cure rate of lymphoma patients has improved, patients’ response to therapy varies widely depending on the types of lymphoma and patient characteristics, ranging from progressive disease to CR [5,20]. The IPI scores of 0–1, 2, 3, and 4–5, developed in the pre-rituximab era using CT and bone marrow assessments, correspond to CR rates of 87%, 67%, 55%, and 44%, respectively [7]. However, its predictive accuracy and clinical utility for treatment response have not been thoroughly assessed in the context of modern therapeutic and imaging approaches. Moreover, while revised indices such as R-IPI and NCCN-IPI improve survival prediction, they did not estimate response rates across risk groups [8,9]. Furthermore, the IPI tool fails to capture the wide range of clinical factors and biomarkers. More importantly, addition of molecular abnormality adds significant value to the prognostication of lymphomas [21]; however, most exiting tools do not have this incorporated necessitating further updated tools which again needs to be tested in bigger cohorts. Hence, an updated risk stratification model incorporating routinely collected clinical variables and biomarkers is needed.

Several studies have demonstrated that inflammatory and nutritional indicators are closely related to the prognosis of cancer patients [12,13,22,23,24,25,26,27,28,29]. Systemic inflammation and inadequate diet promote the proliferation of tumor cells, provide nutrition for tumor cells, stimulate cell growth, and disrupt the immune system, which in turn leads to poor prognosis [30]. Body mass index (BMI), serum albumin, and the prognostic nutritional index (PNI) are often used to assess nutritional status in cancer patients. Traditional inflammatory parameters such as the platelet-to-lymphocyte ratio (PLR), neutrophil-to-lymphocyte ratio (NLR), and lymphocyte-to-monocyte ratio (LMR), along with novel indicators like the systemic immune-inflammation index (SII) and systemic inflammation response index (SIRI) are simple measures to assess systemic inflammation. An increasing amount of research indicates that these inflammation and nutritional indicators are independent predictors of lymphoma prognosis [13,26,27,28]. For example, Liu et al., [12] demonstrated that a nomogram incorporating inflammatory-nutritional markers (SII and PNI) exhibited superior discriminative ability compared to the IPI and NCCN-IPI in predicting OS for DLBCL. Moreover, other studies showed that PNI and SII were significantly associated with complete remission rate [26,28]. However, these studies have only drawn associations between these inflammatory-nutritional indicators and treatment response, without demonstrating whether combining these markers with other prognostic factors enhances predictive accuracy and clinical utility. Although inflammation-nutritional indicators are routinely collected, and relatively inexpensive, their prognostic value in predicting treatment response has been limited in patients with lymphoma.

The pretreatment prediction of therapy response is essential for stratifying patients by their likelihood of achieving a CR and the delivery of precise treatment [31]. However, current clinical prediction tools for forecasting treatment response in lymphoma remain limited. Wang et al. developed a nomogram that integrates imaging features with clinico-pathological factors to assess the CR to chemotherapy in patients with gastric DLBCL [20]. However, this model is not applicable for predicting treatment response before starting therapy, as it was constructed based on post-treatment indicators. Therefore, this study aims to develop and validate a novel prognostic model incorporating pretreatment inflammation-nutritional indicators and using machine learning (ML) for treatment response in B-cell NHL. Additionally, the study evaluated the predictive performance of the IPI, R-IPI, and NCCN-IPI in stratifying patients based on treatment response.

## 2. Methods

### 2.1. Data Source and Study Population

The study utilized data from the prospective binational Lymphoma and Related Diseases Registry (LaRDR; https://lardr.org/), a multicentre registry established in 2016 across Australia and New Zealand. Adult patients (≥18 years) with a new diagnosis of lymphoma, chronic lymphocytic leukemia (CLL), or related diseases in accordance with the World Health Organization (WHO) classification (WHO-HAEM3 or WHO-HAEM4, depending on the time of registration) were included in the registry [32,33]. The methodology of the LaRDR has been described in detail elsewhere [34]. In this study, patients diagnosed with B-cell NHL, namely, DLCBL, FL, MCL and Burkitt lymphoma (BL), who had been treated with chemotherapy/immunotherapy were included.

### 2.2. Study Variables Measurement

Treatment response to first-line chemotherapy/immunotherapy was the primary outcome variable of this study. According to the Lugano 2014 criteria [35], treatment response is categorized as complete response (Deauville score 1–3/disappearance of all evidence of disease), partial response (Deauville score reduction from 4–5 to 1–3/decrease in the size of previously abnormal lesions by at least 50%), no response/stable disease (insufficient reduction to qualify for partial response, but also not meeting criteria for progressive disease), and progressive disease (appearance of new lesions or increase in the size of measurable disease by at least 50% of previously involved sites). In this study, treatment response to first-line therapy was dichotomized into CR and incomplete response (partial response, no response/stable disease, or progressive disease).

Pretreatment factors covering sociodemographic characteristics, clinical features, biomarkers, and inflammatory-nutritional indicators, including age, sex, lymphoma subtype, number of extranodal disease sites, ECOG performance status, B-symptoms, presence of bulk disease (>5 cm), lactate dehydrogenase (LDH), albumin, bilirubin, BMI, C-reactive protein, serum β2 microglobulin, creatinine, alkaline phosphatase (ALP), calcium, hemoglobin, white blood cell count, NLR, MLR, PLR, PNI, SII, SIRI, BCL6 expression and BCL2 expression were considered as potential prognostic factors.

Performance status was measured according to the ECOG (Eastern Cooperative Oncology Group) scale based on four criteria which has been found to be highly correlated with survival and may help predictability to tolerate therapy. BMI is categorized based on WHO cutoff points [36]: underweight (BMI < 18.5 kg/m^2^), normal weight (BMI between 18.5–24.9 kg/m^2^), overweight (BMI between 25–29.9 kg/m^2^), and obese (BMI ≥ 30 kg/m^2^). Patients with anemia and hypoalbuminemia were categorized as per local values and criteria. NLR, MLR and PLR were determined by dividing the absolute counts of neutrophils, monocytes, and platelets by the absolute lymphocyte count, respectively. PNI was expressed as (Albumin (g/L) + 5) × total lymphocyte count  ×  10^9^/L [26]. SII was calculated as neutrophil counts × platelet counts/lymphocyte counts [37]. SIRI was calculated as monocyte count (10^9^/L) × neutrophil count (10^9^/L)/lymphocyte count (10^9^/L) [38].

### 2.3. Statistical Analysis

Patients characteristics were summarized using frequencies and percentages, according to treatment responses (complete versus incomplete), and the Pearson chi-square (χ^2^) test was employed. Receiver operating characteristic (ROC) curve analysis was used to determine the optimal predictive cutoff values for quantitative variables, including ALP, creatinine, bilirubin, NLR, PLR, MLR, PNI, SII, and SIRI. Since missingness in the dataset ranged from 0.07% to 62.4% (Supplementary Table S1), complete case analyses were utilised on variables with less than 5% missing values, while variables with 5% to 40% missing values were handled by multiple imputations using the mice R package. Variables with more than 40% missing values, including C-reactive protein (CRP) and beta-2 microglobulin (B2M), were excluded. All data management and statistical analyses were performed using R version 4.4.2.

### 2.4. Model Development

The data were randomly divided into a training cohort (n = 2221) for tuning model parameters and a validation cohort (n = 553) for predicting model performance metrics (for internal validation) at a 4:1 ratio. The chi-square test was applied to compare the differences in characteristics of patients in the training and validation sets. Six widely used ML algorithms, such as gradient boosting (GBM), K-nearest neighbor (KNN), random forest (RF), support vector machine (SVM), Naïve Bayes (NB), and extreme gradient boosting (XgBoost), along with logistic regression (LR) were employed to predict treatment response of patients with B-cell NHL. The R packages gbm, caret, randomForest, e1071, glmnet, and xgboost were utilized to implement these models. To mitigate the risk of overfitting, a 10-fold cross-validation method was employed in the model training process. In this procedure, the dataset is randomly partitioned into 10 equal folds. Then, the model is trained 10 times, with each iteration using 9 folds for training and the remaining fold for validation and the model performance is averaged across the folds. This approach provides a more reliable estimate of out-of-sample performance compared with a single train test split.

### 2.5. Feature Selection

To select relevant features and achieve efficient data reduction, we employed two stages of variable selection. First, a Boruta algorithm [39,40] was employed to examine the multivariable relationships among the variables, considering all features relevant to the outcome variable. It is a wrapper method built around Random Forests that identifies all features relevant to an outcome rather than only a minimal optimal subset. It works by creating shadow variables (randomly permuted copies of the original features) and then comparing the importance of each real variable to these shadow features. Then, it classifies variables as confirmed (those that outperform the best shadow feature), tentative (those with intermediate performance), or rejected (those that perform worse than the best random shadow feature). Tentative variables whose importance scores are too close to those of shadow features were further evaluated using importance scores to determine inclusion. Secondly, multivariable LR and RF algorithm were employed for variables retained by the Boruta algorithm. The LR model helps identify potential relationships and key variables influencing the outcome [41]. The RF algorithm aids in feature selection by evaluating the importance of variables. To evaluate variable importance, the mean decrease in the Gini index was used. It is a measure of node impurity used in decision trees and Random Forests. It reflects the probability that a randomly chosen observation from a dataset would be incorrectly classified if it were labelled according to the distribution of classes in that node. A Gini index of 0 indicates perfect purity (all cases in the node belong to a single class), while higher values indicate greater heterogeneity. In Random Forests, variable importance is estimated by averaging the reduction in the Gini index each time a variable is used to split the data across all trees in the forest. Variables that achieve larger decreases in impurity are considered more important for prediction [41,42].

### 2.6. Class Imbalance Management

In classification models, ML algorithms often achieve high accuracy for the majority class but assign less importance to the minority class. This imbalance can significantly affect the performance of classifiers. To address class imbalance, we used oversampling, undersampling, and two hybrid methods, such as the Random oversampling of examples (ROSE) and Synthetic minority oversampling (SMOTE) techniques in the training dataset. SMOTE and ROSE are a widely used method to address class imbalance in classification tasks. SMOTE generates synthetic examples of the minority class by interpolating between existing minority instances and their nearest neighbours [43]. ROSE generates synthetic balanced samples by drawing new examples from a smoothed bootstrap distribution of both classes, improving classifier performance in binary imbalanced learning [44].

### 2.7. Model Performance Evaluation

**Figure 1:**
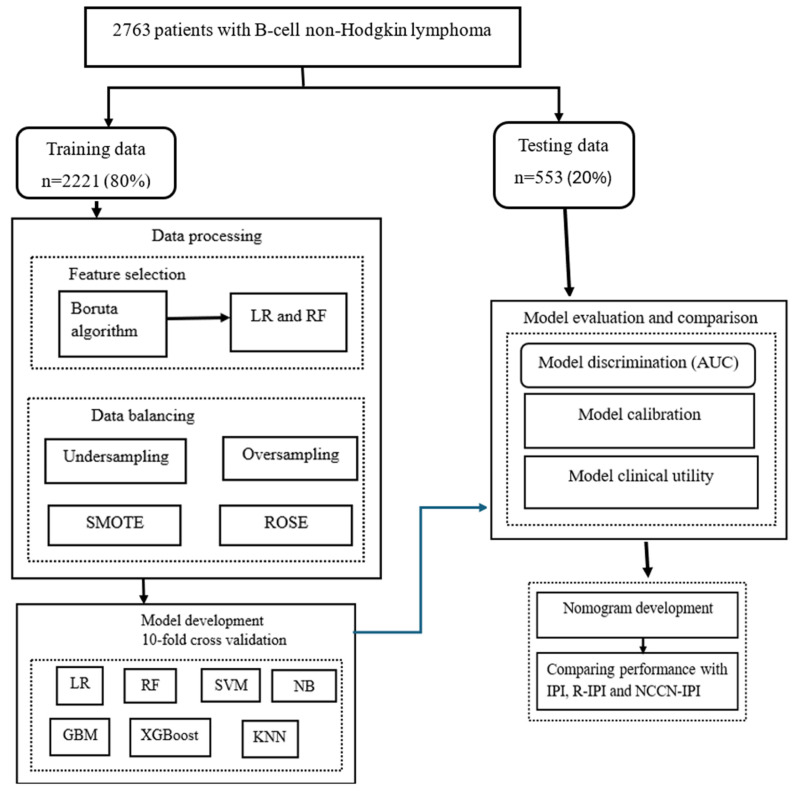
Conceptual framework of the machine learning model development for predicting treatment response of B-cell non-Hodgkin lymphoma. IPI; International Prognostic Index, R-IPI; Revised-IPI, NCCN-IPI; National Comprehensive Cancer Network-IPI, LR; logistic regression, RF; random forest, XgBoost; extreme gradient boosting, KNN; K-nearest neighbour, GBM; gradient boosting, SVM; support vector machine, NB; Naïve Bayes, ROSE; Random oversampling of examples technique, SMOTE; Synthetic minority oversampling technique.

Model discrimination metrics such as the area under the curve (AUC) and classification metrics, including accuracy, sensitivity, specificity, positive and negative predictive values were calculated for each algorithm. The AUC is a discriminating performance indicator that indicates how well a model can distinguish event individuals (i.e., with incomplete response) from non-event individuals (i.e., CR). It ranges from 0 to 1, where values of 0, 0.5 and 1, indicate perfect anti-discrimination, no discrimination, and perfect discrimination, respectively. Brier scores were also used to evaluate the overall agreement between predicted and actual treatment response probabilities, with lower values indicating better calibration and accuracy [45]. In addition, to evaluate and compare developed prediction models in the context of clinical decision-making, a decision curve analysis (DCA) was employed. Based on selected variables, a nomogram for predicting the treatment response of B-cell NHL patients was constructed. This nomogram is a visual tool derived from a statistical model that enables clinicians to estimate the likelihood of a particular clinical outcome. It displays variables separately and assigns to each variable a specific score based on its impact on the probability of the event of interest. By assigning different weights to each risk factor, the nomogram provides a more individualized and accurate risk assessment. The overall score is obtained by summing up the individual variables’ scores [46,47]. Furthermore, the predictive ability of our model, IPI, R-IPI, and NCCN-IPI was compared by AUC, Brier score, and DCA (Figure 1). The R packages pROC, pec, rmda, and rms were used to calculate the AUC, compute the Brier score, perform the DCA, and construct a nomogram, respectively.

## 3. Results

### 3.1. Determination of Cut-Off Values for Inflammatory Nutritional Indicators

A total of 2763 patients with B-cell NHL were included in this study. Using ROC curve, the optimal cut-off points for PLR, MLR, NLR, PNI, SII, and SIRI were 274.773, 0.611, 5.123, 40.93, 1686.985, and 3.529, respectively, with corresponding AUC values of 0.555, 0.563, 0.574, 0.572, 0.570, and 0.574 (Supplementary Table S2). According to the ROC cut-off value, creatinine, bilirubin, ALP, NLR, MLR, PLR, PNI, SII and SIRI were divided into a low and high group.

### 3.2. Background Characteristics

Incomplete response to first-line therapy was more common in patients with adverse clinical features, including stage III/IV, ECOG performance status > 1, elevated LDH, anemia, multiple extranodal sites involvement, and low albumin. Regarding inflammatory nutritional indicators, an incomplete response was more common in patients with low PNI and high SIRI, PLR, NLR, and MLR (Table 1). Additionally, univariable logistic regression identified several factors significantly associated with incomplete response, including advanced stage, poor performance status, elevated LDH, anemia, and inflammatory markers (PNI, SII, SIRI, MLR, PLR, and NLR) (Supplementary Table S3). The training and validation sets were comparable in terms of key characteristics (Supplementary Table S4).

### 3.3. Features Selection

**Figure 2:**
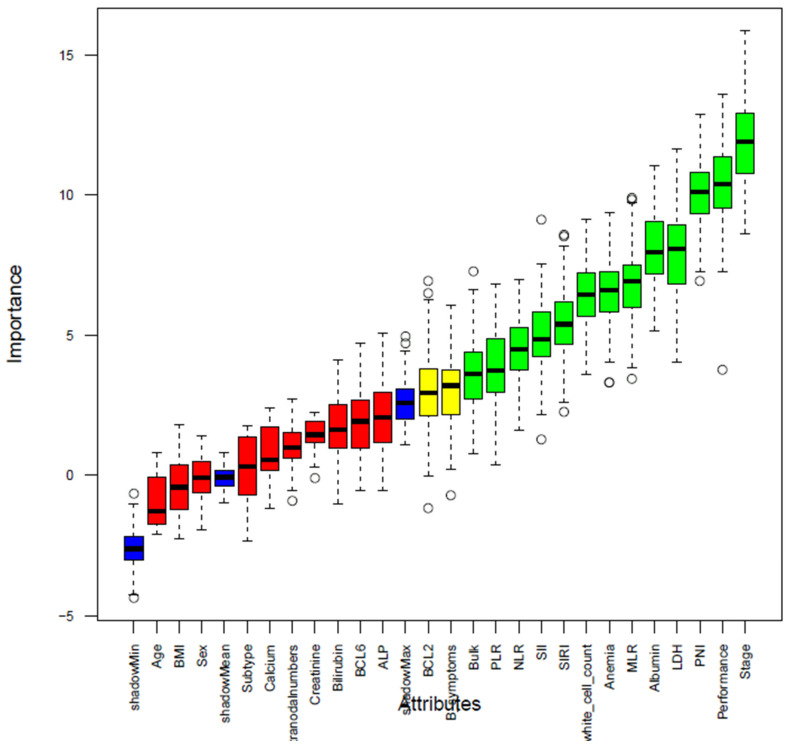
Boruta algorithm feature selection. Each bar represents a variable’s importance score relative to shadow features. Green bars indicate confirmed important variables; yellow indicates tentative; and red indicates rejected variables.

In the first step, based on the Boruta algorithm, out of 25 attributes, 10 were rejected (red boxplots), 13 were confirmed (green boxplots), and 2 were designated as tentative (yellow boxplots) (Figure 2). Both tentative variables were retained (considered as important variables) based on importance scores (Supplementary Table S5). Of the 15 variables retained by the Boruta algorithm, multivariable LR identified six significant independent prognostic factors: performance status, stage, LDH, BCL2 expression, anemia and SII (Supplementary Table S6). The top six important variables from the RF algorithm, including absolute white cell count, bulk, stage, LDH, performance status and BCL2 expression, were identified to compare with the LR model results (Supplementary Figure S1). The combined methods selected eight variables: six significant variables from the LR model, plus additional two factors (bulk disease and white blood cell counts) from RF ranking.

### 3.4. Model Development and Performance

**Figure 3:**
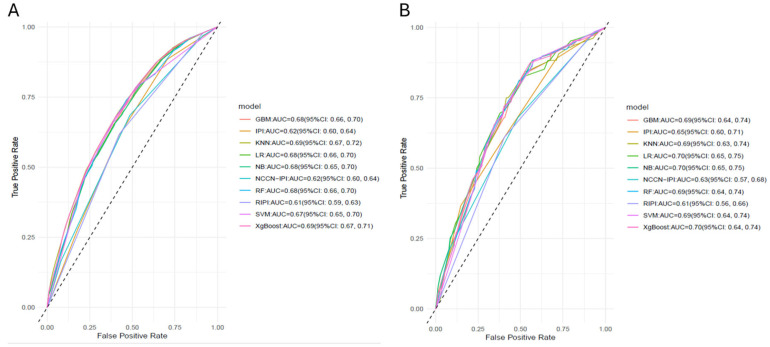
Area under the curve (AUC) for machine learning algorithms and existing prognostic tools: (**A**) training data set, (**B**) Validation set. IPI; International Prognostic Index, R-IPI; Revised-IPI, NCCN-IPI; National Comprehensive Cancer Network-IPI, LR; logistic regression, RF; random forest, XgBoost; extreme gradient boosting, KNN; K-nearest neighbour, GBM; gradient boosting, SVM; support vector machine, NB; Naïve Baye.

In our study, we conducted a comparative analysis of seven algorithms using two distinct predictor sets: variables identified as statistically significant through multivariable LR, and a combination of top six variables based on RF importance with those statistically significant in multivariable LR. Additionally, we employed four methods for managing data imbalance as disproportion in treatment response was encountered, with 75.6% of patients achieving a complete response and 24.4% not achieving a CR. Data balancing improved the performance of only the SVM and RF algorithms, and there was no superior data balancing method, as the performance was consistent across the four data balancing methods (Supplementary Table S7). The inclusion of top variables based on RF importance, alongside those statistically significant in multivariable LR, did not yield a significant enhancement in the predictive performance of the models (Supplementary Figure S2). Consequently, we used six prognostic factors identified as significant through multivariable LR, namely, ECOG performance status, stage, LDH, BCL2 expression, anemia and SII to predict treatment response. The AUCs of all ML in the validation cohort were similar, ranging from 0.69 to 0.70 (Table 2 and Figure 3). The AUC values for all algorithms in both the training and validation sets exhibit minimal differences, indicating that the models were not overfitted and could generalize effectively to unseen data. Moreover, the Brier score ranged from 0.223 for NB to 0.298 for RF, with a score of 1 indicating the poorest calibration and a score of 0 representing perfect calibration (Table 2).

### 3.5. Nomogram Development

**Figure 4:**
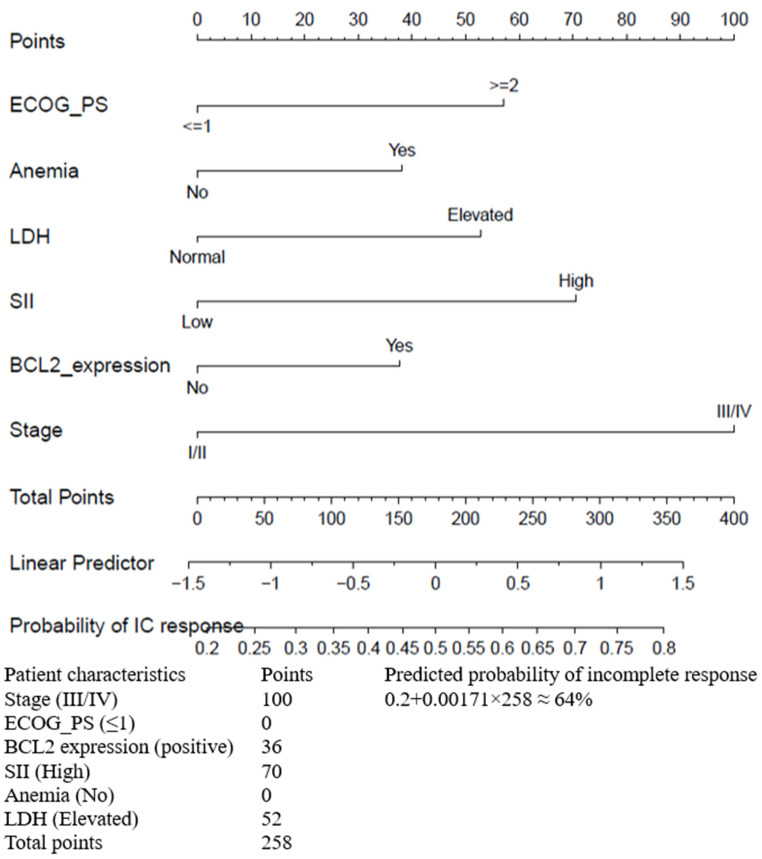
Nomogram with personalized working example for predicting incomplete (IC) treatment response in B-cell lymphomas. ECOG_PS, Eastern Cooperative Oncology Group Performance Status, stage; LDH; Lactate Dehydrogenase; SII, Systemic Immune-Inflammation Index.

The final six independent prognostic factors were integrated into the nomogram to predict treatment response. Variable scores can be obtained by where the vertical line intersects the point scale at the top of the chart for each variable (Figure 4), and these scores can then be summed up to get a total score. This total score provides predictive measures of treatment response for each patient. The bottom of the series shows the model’s estimated probability of incomplete response after chemotherapy/immunotherapy in patients with B-cell NHL. For example, a patient with performance status (≥2), anemia, elevated LDH, high SII, positive BCL2 expression, and stage III/IV could obtain 57 points, 37 points, 52 points, 70 points, 36 points, and 100 points, respectively, resulting in a total score of 352 points. For patients having all these six risk factors, their probability of having an incomplete response is 80%, compared to 20% for patients without these six risk factors (Figure 4). Generally, the probability of an incomplete (IC) response can be approximated using the formula:

Probability of IC response = 0.2 + 0.00171 × Total points, where 0.0017 is the slope (scaling factor).

**Figure 5:**
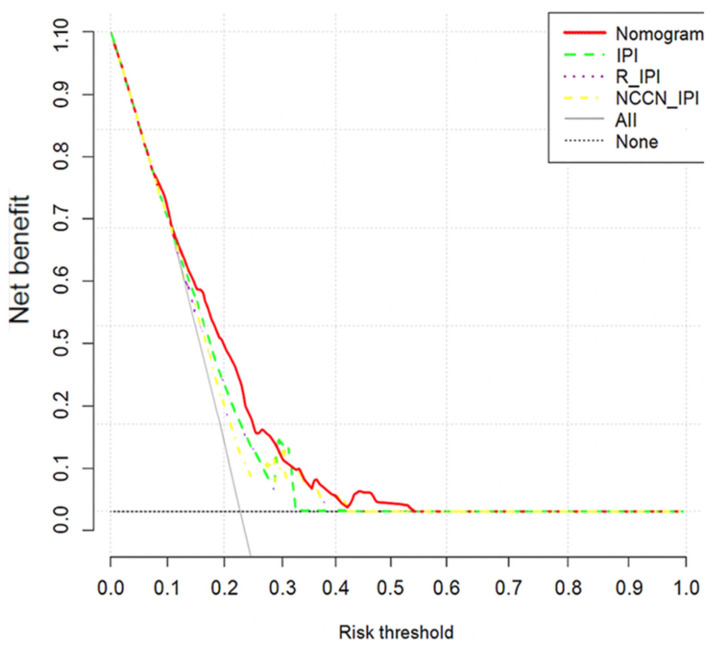
Decision curve analysis of nomogram and existing prognostic tools. IPI; International Prognostic Index, R-IPI; Revised-IPI, NCCN-IPI; National Comprehensive Cancer Network-IPI.

The AUCs for the nomogram in the validation cohort was 0.70 (95% CI: 0.65, 0.75), indicating that our model had acceptable discriminating ability. The AUC of the nomogram outperformed the Revised IPI (AUC = 0.61, 95% CI: 0.56, 0.66), NCCN-IPI (AUC = 0.63, 95% CI: 0.57, 0.68), and IPI (AUC = 0.65, 95% CI: 0.60, 0.71). Calibration of the nomogram was superior, with a lower Brier score (0.227) compared to existing indices (Table 2). Furthermore, the decision curve analysis confirmed the clinical utility was better for nomogram (Figure 5).

Based on quantiles, the total score of the nomogram was categorized into four risk groups: low risk (<138 points), low intermediate (138–188 points), high intermediate (188–225 points) and high-risk (≥225 points). In the validation cohort, there were 218, 125, 93, and 117 patients in the low-risk, low intermediate, high intermediate, and high-risk groups, respectively, with corresponding incomplete response rates of 9.1%, 25.6%, 32.3%, and 39.3% (Table 3).

As the IPI, R-IPI, and NCCN-IPI were originally developed for prognostication in DLBCL, we evaluated their performance specifically within the DLBCL cohort to ensure a fair comparison and to further contextualize their utility. In the validation cohort, our unified model demonstrated slightly superior discriminative ability compared to IPI, R-IPI, and NCCN-IPI (AUC: 0.64 vs. 0.59, 0.57, and 0.60, respectively) (Supplementary Figure S3). These findings suggest that our model may offer slightly improved predictive accuracy, even within the subtype for which these existing clinical scores were originally designed.

## 4. Discussion

Here, we developed and internally validated a nomogram and machine learning algorithms using universally collected clinical features, biomarkers, and inflammatory-nutritional markers data from 33 hospitals to assess the predictability of treatment response. In this study, both LR and ML (RF) identified overlapping key predictors, including stage, LDH, ECOG performance status, and BCL2 expression. Overall, ML algorithms and LR analysis demonstrated comparable predictive ability in predicting treatment response. Notably, the nomogram showed significantly better discriminative ability compared to the R-IPI.

In this study, the comparable predictive performance observed between ML algorithms and LR can be attributed to several factors. First, although categorizing continuous variables are not a statistically recommended practice in predictive modelling [48,49], we categorized them based on predefined cutoff points or ROC curve analysis for simplicity in clinical use. Categorizing continuous variables may have influenced ML model performance. For example, categorization can lead to a loss of information and reduce their ability to capture complex, non-linear relationships, particularly in tree-based methods. Second, the number of predictors used in model development was limited. It is noted that standard regression methods perform well when applied to datasets with relatively few predictor variables and large sample sizes [50]. While no definitive threshold exists for the number of predictors required to enhance ML performance, incorporating more variables may have provided ML algorithms with a more significant advantage over LR.

Although the application of ML in predicting healthcare outcomes has been increasing in recent years, evidence regarding its superiority over traditional regression methods remains inconclusive [51,52,53,54,55]. Consistent with our findings, a registry-based study from the European Society for Blood and Marrow Transplantation reported that LR performed comparably to ML algorithms in predicting hematopoietic stem cell transplantation-related mortality in patients with acute leukemia [51]. Similarly, studies utilizing electronic health records (EHR) in China found no significant difference in the discriminatory ability of ML models and LR for predicting recurrence and mortality in patients with DLBCL [52,53]. Similar performances of LR and ML have also been reported for predicting solid tumors and non-oncologic outcomes, for example, predicting gastric cancer risk [55] and hypertension incidence [56]. In contrast, an EHR-based study from Shanxi Tumor Hospital demonstrated that ML outperformed LR in stratifying recurrence risk among DLBCL patients [54]. Moreover, a systematic review and meta-analysis demonstrated the superiority of ML in predicting overall survival in lung cancer [57] and treatment response in rectal cancer patients [58]. These inconclusive results suggest that further investigation is needed to understand the conditions under which ML models outperform traditional methods.

Given the comparable performance of LR and ML, we developed an LR-based nomogram to aid in incorporating our model into clinical practice. It is important to note that although our nomogram’s AUC of 0.70 appear modest, it exceeds that of well-established prognostic indices, including the IPI (0.65), R-IPI (0.61), and NCCN-IPI (0.63). To our knowledge, only one previous study has developed a nomogram for predicting treatment response in lymphoma [20]. Although the nomogram demonstrated promising discrimination (AUC = 0.957) in gastric DLBCL, its application may be limited by a small sample size (n = 108 patients), a single-centre design, dependence on imaging features not routinely available in practice and reliance on post-treatment indicators, which restrict its use for pre-treatment risk stratification. Other studies have also investigated the prediction of treatment response in lymphoma subtypes, such as primary central nervous system lymphoma and bulky Hodgkin and non-Hodgkin lymphomas, using advanced imaging or radiomic features, with a small sample size from single-centre settings. In these studies, discrimination has ranged from AUC 0.618 to 0.868, with combined radiomics–clinical models often performing better than clinical models alone [59,60]. While such studies suggest potential value in incorporating radiomics, our recent meta-analysis demonstrated that models incorporating radiomic features performed similarly to models based on clinical features in hematological malignancies [61]. In our multilevel meta-analysis of 38 ML models developed for lymphoma outcomes, we observed a pooled AUC of 0.779, which was higher than the current study’s performance, with most studies predicting OS and PFS. Taken together, our nomogram derived from routinely collected pre-treatment variables across 33 hospitals enables early prediction before therapy initiation, offers substantial potential for generalizability, and remains feasible even in resource-limited settings.

Importantly, although the AUC is a fundamental metric for assessing model discrimination, it does not capture a model’s calibration or its clinical usefulness. Model’s clinical utility can be assessed using DCA by evaluating the net benefit across different threshold probabilities [62,63]. DCA demonstrated that the nomogram provided a better net benefit over IPI-based scoring systems across a range of threshold probabilities, showing better clinical utility for guiding treatment decisions. Moreover, the Brier score for our nomogram was lower than that of existing prognostic indices, suggesting better agreement between predicted and observed probabilities, thereby providing reliable risk estimation. In our study, four risk groups were categorized based on total nomogram scores derived from ROC curve analysis rather than assigning equal weight to all factors as used in IPI-based scoring systems. Assigning equal weight to all factors can lead to a loss of discrimination power and inaccurate stratification since not all risk factors have an equal impact on the occurrence of an outcome. Our approach addressed this methodological limitation by allowing for more precise and detailed individualized risk stratification. Additionally, our nomogram included BCL2 expression, anemia, and SII, which were not originally part of the IPI-based scoring systems. This demonstrates the slightly enhanced predictive power and clinical usefulness of our model in stratifying patients.

In contrast to disease-specific prognostic indices such as the IPI for DLBCL, FLIPI for FL, and M-IPI for MCL, our model offers a unified prognostic tool applicable across multiple B-cell lymphoma subtypes. Although these existing indices were developed within subtype-specific cohorts, they primarily rely on general clinical parameters such as LDH, age, performance status, stage, extranodal involvement, and blood counts, which are not disease-specific markers but rather indicators of overall tumor burden and patient condition. Our model includes some of the shared factors found across these existing indices namely LDH, stage, and ECOG performance status and incorporating hemoglobin, which is included in FLIPI but not in IPI or MIPI. We also evaluated white blood cell count, a component of MIPI, as an independent variable, though it did not retain significance in the final model. Importantly, our model extends beyond conventional clinical variables by integrating BCL2 expression, a marker of anti-apoptotic signaling and treatment resistance [64,65] and the SII, a composite biomarker reflecting the host’s immune and inflammatory status [66]. Notably, the lymphoma subtype was not a significant predictor of initial treatment response in our cohort, suggesting that the features included in our model may capture shared biological and clinical characteristics relevant to treatment response across subtypes despite their known heterogeneity. As such, our model may serve as a practical and unified tool to stratify treatment response in various B-cell lymphomas. To further ensure fair comparison and clinical relevance, we propose to evaluate the model’s performance within individual subtypes using larger cohorts and benchmarking it against established disease-specific indices such as the FLIPI, and MIPI.

The prognostic significance of variables incorporated in our nomogram, including stage, LDH, ECOG performance status, BCL2 expression, anemia, and SII, has been well-documented in cancer prognosis [7,8,9,67,68,69,70,71]. Apart from factors included in the IPI, increasingly more evidence suggests that inflammatory-nutritional markers play a significant role in lymphoma prognosis [12,13,26,27,28]. We comprehensively tested various inflammatory-nutritional markers and found that only the SII was a significant independent risk factor for treatment response. The SII, a comprehensive inflammatory biomarker that incorporates neutrophil, platelet, and lymphocyte counts, was developed in 2014 to predict poor outcomes in patients with hepatocellular carcinoma. It is linked to circulating tumor cells and reflects the balance of the body’s inflammatory and immune responses, with higher SII values linked to poorer patient outcomes [66]. Our study observed that high SII were associated with a higher rate of incomplete responses in B-cell lymphoma patients. Consistent with our findings, a prospective study by Waley et al. [28] demonstrated that patients with high SII was significantly associated with low CR rate in patients with DLBCL. The prognostic role of SII in predicting survival outcomes for patients with lymphoma has also been well-established [12,13]. In addition, numerous meta-analyses have confirmed that high SII is associated with worse prognoses in a variety of tumors [72,73], as well as poor cardiovascular outcomes and an increased risk of cardiovascular diseases [74,75].

Moreover, our study demonstrated that patients with pretreatment anemia were more likely to have incomplete responses to treatment. Notably, anemia is a key component of the FLIPI score and has been reaffirmed as a significant prognostic factor in a recent model, the FL Evaluation Index (FLEX) [10,76]. Moreover, studies have shown that pretreatment anemia provides additional predictive value to the IPI, R-IPI, and NCCN-IPI in predicting OS for patients with DLBCL [77,78]. This can be attributed to hypoxia induced by low levels of hemoglobin, which has been shown to contribute to tumor progression and therapy resistance by promoting angiogenesis, inducing genomic mutations, and increasing resistance to apoptosis and the cytotoxic effects of chemo/radiotherapy-generated free radicals [79,80]. Given that anemia is a common hematologic abnormality in cancer patients and can be further induced by chemotherapy [81,82], our study suggests that incorporating pretreatment anemia status into existing prognostic tools for lymphoma patients may enhance their accuracy. This could help tailor treatment strategies more effectively, thereby improving therapy response and overall prognosis.

Furthermore, building on the NCCN-IPI recommendation that the inclusion of biological markers such as BCL2 expression may enhance prognostic accuracy [9], we tested the significance of BCL2 expression and found that positive BCL2 expression was significantly associated with a higher rate of incomplete treatment responses. Similarly, in previous studies, positive BCL2 expression has been associated with an increased risk of recurrence, poor treatment response, and shorter PFS and OS in lymphoma patients [83,84,85,86], as well as poor therapy response in acute leukemia [87]. This finding aligns with the general understanding that overexpression of BCL2 can lead to the survival of abnormal cells that should otherwise undergo apoptosis, thereby contributing to tumor growth and resistance to therapy [64,65]. On the contrary, a recent study has shown that patients with BCL2 dependence in chronic lymphocytic leukemia (CLL) tend to respond favorably to therapy [88]. While BCL2 overexpression generally indicates poor prognosis due to enhanced cell survival and resistance to apoptosis, these findings underscore the complex role of BCL2 in cancer prognosis and warrant further investigation.

This study has some strengths and limitations. To the best of our knowledge, this is the first comprehensive comparison of various machine learning algorithms alongside standard regression techniques, as well as the validation of existing tools for predicting treatment response in B-cell lymphomas. Additionally, the utilization of multicenter data enhances the diversity and representativeness of the patient cohort. Although the model was developed using a multi-institutional dataset, and treatment response did not significantly differ across subtypes, the predominance of DLBCL cases may still influence our unified model’s performance. While we validated the model specifically in DLBCL, comparing its performance with IPI, R-IPI, and NCCN-IPI, further validation in rarer subtypes is needed to confirm broader applicability. Moreover, the study’s reliance on internal validation limits its generalizability, underscoring the need for external validation. In addition, although ROC curve analysis was employed to determine optimal predictive cutoff values for inflammatory-nutritional markers, an advantageous method for evaluating model performance across different thresholds, these cutoff points may not be universally applicable across diverse populations or clinical settings. Therefore, future research should prioritize the validation of these cutoff points to ensure their generalizability and reliability in varied clinical settings.

## 5. Conclusions

In conclusion, our study developed and internally validated predictive models for treatment response in lymphoma patients, demonstrating that machine learning models and standard regression have comparable performance. Although our nomogram, which incorporates clinical (stage, LDH, anemia and ECOG performance status), inflammatory (SII), and molecular (BCL2 expression) features, demonstrated slightly improved discriminative ability and clinical utility compared to existing tools, the overall discriminatory power remains limited. To advance prognostic accuracy and better reflect the evolving landscape of lymphoma care, future work will focus on integrating additional molecular parameters and transcriptomic signatures. Moreover, as our model is only internally validated, external validation is warranted to confirm its generalizability. This supports its potential use in risk stratification and decision-making for tailored treatment strategies in managing lymphoma patients.

## Figures and Tables

**Table 1:** Treatment response according to patient characteristics.

Variables	Treatment Response, N (%)		X^2^ (*p*-Value)
	Complete	Incomplete	
Sex			
Male	1231 (74.9)	412 (25.1)	0.93 (0.334)
Female	858 (76.6)	262 (23.4)	
Age			
≤60	675 (77.3)	198 (22.7)	1.90 (0.168)
>60	1414 (74.8)	476 (25.2)	
BMI			
Underweight	47 (72.1)	19 (28.8)	6.91 (0.075)
Normal	668 (73.1)	246 (26.9)	
Overweight	726 (76.1)	228 (23.9)	
Obese	648 (78.2)	181 (21.8)	
Stage			
I	359 (89.5)	42 (10.5)	84.89(<0.001)
II	330 (83.8)	64 (16.2)	
III	402 (75.4)	131 (24.6)	
IV	998 (69.5)	437 (30.5)	
I or II	689 (86.7)	106 (13.3)	73.19 (<0.001)
III or IV	1400 (71.1)	568 (28.9)	
Subtype			
DLBCL	1480 (74.1)	518 (25.9)	9.63 (0.022)
FL	412 (79.1)	109 (20.9)	
MCL	144 (80.0)	36 (20.0)	
BL	53 (82.8)	11 (17.2)	
ECOG performance status			
0 or 1	1863 (78.0)	525 (22.0)	54.40 (<0.001)
2–4	226 (60.3)	149 (39.7)	
LDH			
Normal	1109 (83.1)	225 (16.9)	78.45 (<0.001)
Elevated	980 (68.6)	449 (31.4)	
B symptoms			
Absent	1686 (77.1)	500 (22.9)	12.74 (<0.001)
Present	403 (69.8)	174 (30.2)	
BCL6 expression			
Negative	636 (73.8)	226 (26.2)	2.12 (0.14)
Positive	1453 (76.4)	448 (23.6)	
BCL2 expression			
Negative	758 (78.5)	207 (21.5)	6.72 (0.009)
Positive	1331 (74.0)	467 (26.0)	
Number of Extranodal sites			
≤1	1419 (77.9)	402 (22.1)	15.19 (<0.001)
>1	670 (71.1)	272 (28.9)	
Bulk disease			
No	1392 (78.2)	388 (21.8)	17.89 (<0.001)
Yes	697 (70.9)	286 (29.1)	
Anemia			
No	1317 (81.2)	304 (18.8)	66.90 (<0.001)
Yes	772 (67.6)	370 (32.4)	
Albumin			
Low	662 (67.5)	319 (32.5)	53.75 (<0.001)
High	1427 (80.1)	355 (19.9)	
Creatinine			
Low (≤95.5)	1674 (76.4)	516 (23.6)	3.75 (0.053)
High (>95.5)	415 (72.4)	158 (27.6)	
Alkaline phosphate			
Low (≤83.5)	1090 (78.5)	298 (21.5)	12.61 (<0.001)
High (>83.5)	999 (72.7)	376 (27.3)	
Bilirubin			
Low (≤40.5)	2052 (75.9)	653 (24.1)	3.85 (0.050)
High (>40.5)	37 (63.8)	21 (36.2)	
PNI			
Low (≤40.93)	626 (67.8)	297 (32.2)	44.90 (<0.001)
High (>40.93)	1463 (79.5)	377 (20.5)	
SII			
Low (≤1686.985)	1651 (79.1)	436 (20.9)	55.97 (<0.001)
High (>1686.985)	438 (64.8)	238 (35.2)	
SIRI			
Low (≤3.529)	1542 (79.2)	406 (20.8)	44.52 (<0.001)
High (>3.529)	547 (67.1)	268 (32.9)	
MLR			
Low (≤0.611)	1479 (79.0)	394 (21.0)	34.99 (<0.001)
High (>0.611)	610 (68.5)	280 (31.5)	
PLR			
Low (≤274.773)	1561 (78.8)	419 (21.2)	38.96 (<0.001)
High (>274.773)	528 (67.4)	255 (32.6)	
NLR			
Low (≤5.123)	1503 (79.3)	397 (20.7)	43.40 (<0.001)
High (>5.123)	586 (67.6)	281 (32.4)	
IPI risk group			
Low	625 (89.0)	77 (11.0)	117.27 (<0.001)
Low intermediate	592 (76.4)	183 (23.6)	
High intermediate	513 (70.4)	216 (29.6)	
High	359 (64.5)	198 (35.5)	
Revised IPI risk group			
Low	159 (91.4)	15 (8.6)	87.97 (<0.001)
Intermediate	1058 (81.2)	245 (18.8)	
High	872 (67.8)	414 (32.2)	
NCCN-IPI risk group			
Low	175 (88.8)	22 (11.2)	106.35 (<0.001)
Low intermediate	919 (82.7)	192 (17.3)	
High intermediate	819 (70.7)	340 (29.3)	
High	176 (59.5)	120 (40.5)	

MLR, monocyte-to-lymphocyte ratio; NLR, Neutrophil-to-Lymphocyte Ratio; PLR, Platelet-to-Lymphocyte Ratio; PNI, Prognostic nutrition index; SII, Systemic Immune-Inflammation Index; SIRI, Systemic Inflammation Response Index; IPI, International Prognostic Index; NCCN-IPI, National Comprehensive Cancer Network-IPI.

**Table 2:** Performance of machine learning algorithms and IPI based scoring systems.

Model	Discrimination and Classification Metrics						Calibration
	AUC	Accuracy	Sensitivity	Specificity	PPV	NPV	Brier Score
IPI	0.65	0.60	0.61	0.59	0.31	0.83	0.235
R-IPI	0.61	0.60	0.61	0.59	0.31	0.83	0.239
NCCN-IPI	0.63	0.55	0.68	0.51	0.30	0.84	0.238
LR	0.70	0.62	0.70	0.60	0.35	0.87	0.227
RF	0.69	0.62	0.73	0.59	0.35	0.88	0.298
XgBoost	0.70	0.61	0.73	0.58	0.34	0.88	0.231
KNN	0.69	0.61	0.76	0.57	0.35	0.89	0.233
GBM	0.69	0.61	0.68	0.60	0.34	0.86	0.232
SVM	0.69	0.62	0.70	0.59	0.34	0.87	0.229
NB	0.70	0.68	0.58	0.69	0.36	0.85	0.223

IPI, International Prognostic Index; R-IPI, Revised-IPI; NCCN-IPI, National Comprehensive Cancer Network-IPI; LR, logistic regression; RF, random forest; XgBoost, extreme gradient boosting; KNN, K-nearest neighbor; GBM, gradient boosting; SVM, support vector machine; NB, Naïve Bayes; PPV, positive predictive value; NPV, negative predictive value.

**Table 3:** Treatment response across risk groups of Models in the validation set.

	Model	Risk Groups (Score)			
	Nomogram	Low (<138)	LI (138–188)	HI (188–225)	High (≥225)
Risk factors and scoring	Stage (I/II = 0; III/IV = 100) ECOG PS (≤1 = 0; >1 = 57) BCL2 Expression (Negative = 0; Positive = 36) SII (Low = 0; High = 70) Anemia (No = 0; Yes = 37) LDH (Normal = 0; Elevated = 52)				
Frequency (%)		218 (39.4)	125 (22.6)	93 (16.8)	117 (21.2)
Treatment response	Complete (%)	90.9	74.4	67.7	60.7
	Incomplete (%)	9.1	25.6	32.3	39.3
	**IPI**	**Low (0–1)**	**LI (2)**	**HI (3)**	**High (4–5)**
Risk factors and scoring	Age (≤60 = 0; >60 = 1) Stage (I/II = 0; III/IV = 1) LDH (Normal = 0; Elevated = 1) ECOG PS (≤1 = 0; >1 = 1) Extranodal Sites (≤1 = 0; >1 = 1)				
Frequency (%)		136 (25.6)	167 (30.2)	140 (25.3)	110 (19.9)
Treatment response	Complete (%)	89.7	78.4	77.9	57.3
	Incomplete (%)	10.3	21.6	22.1	42.7
	**R-IPI**	**Very good (0)**	**Good (1–2)**	**Poor (3–5)**	
Risk factors and scoring	Age (≤60 = 0; >60 = 1) Stage (I/II = 0; III/IV = 1) LDH (Normal = 0; Elevated = 1) ECOG PS (≤1 = 0; >1 = 1) Extranodal Sites (≤1 = 0; >1 = 1)				
Frequency (%)		30 (5.4)	273 (49.4)	250 (45.2)	
Treatment response	Complete (%)	93.3	82.4	68.8	
	Incomplete (%)	6.7	17.6	31.2	
	**NCCN-IPI**	**Low (0–1)**	**LI (2–3)**	**HI (4–5)**	**High (6–8)**
Risk factors and scoring	Age (<40 = 0; 41–60 = 1; 61–75 = 2; >75 = 3) LDH (≤ULN = 0; >ULN–≤3 × ULN = 1; >3 × ULN = 2) Stage (I/II = 0; III/IV = 1) ECOG PS (≤1 = 0; >1 = 1) Major Extranodal Sites (No = 0; Yes = 1)				
Frequency (%)		34 (6.1)	226 (40.9)	232 (42.0)	61 (11.0)
Treatment response	Complete (%)	91.2	83.2	74.1	55.7
	Incomplete (%)	8.8	16.8	25.9	44.3

LI; Low Intermediate, HI; High Intermediate, LDH; Lactate Dehydrogenase, ECOG PS; Eastern Cooperative Oncology Group performance status, IPI; International Prognostic Index, R-IPI; Revised-IPI, NCCN-IPI; National Comprehensive Cancer Network-IPI, SII; Systemic Immune-Inflammation Index.

## Data Availability

The data supporting this study’s results can be obtained from the Lymphoma and Related Diseases Registry (LaRDR) with permission from the Steering Committee, and the request complies with the LaRDR Data Access Policy.

## Supplementary Materials

The following supporting information can be downloaded at: https://www.mdpi.com/article/10.3390/jcm14207445/s1, Table S1: Proportion of missing data for variables; Table S2: Optimal cutoff points and performance metrics for inflammatory nutritional indicators; Table S3: Univariable logistic regression for treatment response; Table S4: Background characteristics of study participants in the training cohort and validation cohort; Table S5: Prognostic factors retained and rejected by final Boruta feature selection methods; Table S6: Multivariable logistic regression in training data dataset; Table S7: Performance of machine learning algorithms according to different data imbalance management methods; Figure S1: Features ranking based on random forest algorithm. LDH, lactate dehydrogenase; MLR, monocyte-to-lymphocyte ratio; Figure S2: Area under the curve (AUC) for machine learning algorithms with features selected using combined methods in the testing data set, balanced with SMOTE; Figure S3: Area under the curve (AUC) for our model (Nomogram) and existing tools in the testing data set for Diffuse large B-cell lymphoma.

## Abbreviations

AUC	Area Under the Curve
CI	Confidence Interval
CR	Complete response
GBM	Gradient Boosting Model
IPI	International Prognostic Index
LaRDR	Lymphoma and Related Diseases Registry
LDH	Lactate Dehydrogenase
LR	Logistic Regression
ML	Machine Learning
MLR	monocyte-to-lymphocyte ratio
NCCN-IPI	National Comprehensive Cancer Network International Prognostic Index
NLR	Neutrophil-to-Lymphocyte Ratio
PFS	Progression Free survival
PLR	Platelet-to-Lymphocyte Ratio
PNI	Prognostic nutrition
RF	Random Forest
R-IPI	Revised International Prognostic Index
SII	Systemic Immune-Inflammation Index
SIRI	Systemic Inflammation Response Index
OS	Overall Survival
