# Supplementary material for: Development and Validation of Prognostic Models for Treatment Response of Patients with B-Cell Lymphoma: Standard Statistical and Machine-Learning Approaches
Source: J Clin Med. 2025 Oct 21;14(20):7445. doi: 10.3390/jcm14207445 (PMC12565506; doi:10.3390/jcm14207445)
Supplement: Supplementary file 1 [file jcm-14-07445-s001.zip › jcm-3910362-supplementary.pdf]

Title: Development and validation of prognostic models for treatment response of patients with B- cell lymphoma: Statistical and machine-learning techniques

Supplementary tables

Supplementary Table S1: Proportion of missing data for variables

Variables	Missing values	Percent
β_2 microglobulin	1839	62.4
C-reactive protein	1838	62.4
Alkaline phosphatase	652	22.1
Creatinine	602	20.4
Bilirubin	586	19.9
ECOG performance status	408	14.2
Bulk disease	295	10.0
BCL2 expression	289	9.8
BCL6 expression	289	9.8
Stage	260	8.8
Lactate Dehydrogenase (LDH)	195	6.6
Calcium	166	5.6
Height	129	4.4
Weight	110	3.7
Albumin	43	1.5
Platelet count	32	1.1
Monocyte count	31	1.0
White cell count	30	1.0
Neutrophil count	30	1.0
Lymphocyte count	30	1.0
Haemoglobin	28	0.9
Sex	2	0.07

Supplementary Table S2: Optimal cutoff points and performance metrics for inflammatory nutritional indicators

Variable	Cut of points	AUC	Accuracy	Sensitivity	Specificity
SII	1686.985	0.570	0.684	0.353	0.790
SIRI	3.529	0.574	0.655	0.398	0.738
PNI	40.93	0.572	0.637	0.441	0.700
MLR	0.611	0.563	0.637	0.415	0.708
PLR	274.773	0.555	0.657	0.378	0.747
NLR	5.123	0.574	0.647	0.417	0.719
ALP	83.5	0.537	0.531	0.558	0.522
Creatinine	95.5	0.508	0.663	0.234	0.801
Bilirubin	40.5	0.500	0.750	0.031	0.982

Note: ALP, Alkaline phosphate; MLR, monocyte-to-lymphocyte ratio; NLR, Neutrophil-to-Lymphocyte Ratio; PLR, Platelet-to-Lymphocyte Ratio; PNI, Prognostic nutrition; SII, Systemic Immune-Inflammation Index; SIRI, Systemic Inflammation Response Index

Supplementary Table S3: Univariable logistic regression for treatment response.

Variables	Crude odds ratio (95% CI)
Sex	
Female	1
Male	1.15(0.94, 1.40)
Age	
≤60	1
>60	1.16(0.94, 1.43)
Body mass index	
Underweight	1
Normal	0.84(0.46, 1.61)
Overweight	0.76(0.41, 1.46)
Obese	0.68(0.37, 1.30)
Stage	
I/II	1
III/IV	2.70(2.11, 3.49)*
Subtype	
Burkitt lymphoma	1
Diffuse large B-cell lymphoma	1.76(0.89, 3.87)
Follicular lymphoma	1.45(0.71, 3.27)
Mantel cell lymphoma	1.25(0.57, 2.99)
ECOG performance status	
0 or 1	1
2-4	2.23(1.73, 2.87)*
LDH	
Normal	1
Elevated	2.13(1.74, 2.61)*
B symptoms	
Absent	1
Present	1.42(1.13, 1.78)*
BCL6 expression	
Negative	1.20(0.98, 1.48)
Positive	1
BCL2 expression	
Negative	1
Positive	1.28(1.05, 1.58)*

Number of Extranodal sites	
≤1	1
>1	1.34(1.10, 1.64)*
Bulk disease	
No	1
Yes	1.42(1.16, 1.73)*
Anemia	
No	1.99(1.63, 2.41)*
Yes	
Albumin	
Low	1.95(1.60, 2.37)*
High	1
Creatinine	
Low (≤95.5)	1
High (>95.5)	1.14(0.90, 1.44)
Alkaline phosphate	
Low (≤83.5)	1
High (>83.5)	1.41(1.16, 1.71)*
Bilirubin	
Low (≤40.5)	1
High (>40.5)	1.59(0.84, 2.89)
Prognostic nutrition	
Low (≤ 40.93)	1.90(1.56, 2.32)*
High (>40.93)	1
Systemic Immune-Inflammation index	
Low (≤ 1686.985)	1
High (>1686.985)	2.09(1.69, 2.58)*
Systemic Inflammation Response Index	
Low (≤ 3.529)	1
High (>3.529)	1.94(1.59, 2.38)*
Monocyte-to-lymphocyte ratio	
Low (≤0.611)	1
High (>0.611)	1.80(1.47, 2.20)*
Platelet-to-Lymphocyte Ratio	
Low (≤274.773)	1
High (>274.773)	1.74(1.41, 2.13)*
Neutrophil-to-Lymphocyte Ratio	
Low (≤5.123)	1
High (>5.123)	1.90(1.56, 2.33)*
White blood cell count	1.001(0.998, 1.004)

Supplementary Table S4: Background characteristics of study participants in the training cohort and validation cohort

Variables	Whole cohort	Training set	Validation set	P-value
	N=2763 (%)	N=2210(%)	N=553(%)	
Sex				
Male	1643(59.5)	1306(59.1)	337(60.9)	0.458
Female	1120(40.5)	904(40.9)	216(39.1)	
Age				
≤60	873(31.6)	695(31.4)	178(32.2)	0.776
>60	1890(68.4)	1515(68.6)	375(67.8)	
Body mass index				
Underweight	66(2.4)	50(2.3)	16(2.9)	0.323
Normal	914(33.1)	748(33.8)	166(30.0)	
Overweight	954(34.5)	753(34.1)	201(36.3)	
Obese	829(30.0)	659(29.8)	170(30.7)	
Stage				
I/II	795(28.8)	638(28.9)	157(28.4)	0.975
III/IV	1968(71.2)	1572(71.1)	396(71.6)	
Subtype				
DLBCL	1998(72.3)	1589(71.9)	409(74.0)	0.705
FL	521(18.9)	422(19.1)	99(17.9)	
MCL	180(6.5)	145(6.6)	35(6.3)	
BL	64(2.3)	54(2.4)	10(1.8)	
Performance status				
0 or 1	2388(86.4)	1898(85.9)	490(88.6)	0.109
2-4	375(13.6)	312(14.1)	63(11.4)	
LDH				
Normal	1334(48.3)	1084(49.0)	250(45.2)	0.270
Elevated	1429(51.7)	1126(51.0)	303(54.8)	
B-symptoms				
Absent	2186(79.1)	1738(78.6)	448(81.0)	0.243
Present	577(20.9)	472(21.4)	105(19.0)	
BCL6 expression				
Negative	862(31.2)	701(31.7)	161(29.1)	0.258
Positive	1901(68.8)	1509(68.3)	392(70.9)	
BCL2 expression				
Negative	965(34.9)	768(34.8)	197(35.6)	0.737
Positive	1798(65.1)	1442(65.2)	356(64.4)	
Number of Extranodal sites				
≤1	1821(65.9)	1449(65.6)	372(67.3)	0.480
>1	942(34.1)	761(34.4)	181(32.7)	
Bulk disease				
No	1780(64.4)	1442(65.2)	338(61.1)	0.078
Yes	983(35.6)	768(34.8)	215(38.9)	
Anaemia				
No	1621(58.7)	1304(59.0)	317(57.3)	0.503
Yes	1142(41.3)	906(41.0)	236(42.7)	
Albumin				
Low	981(35.5)	786(35.6)	195(35.3)	0.933
High	1782(64.5)	1424(64.4)	358(64.7)	

Creatinine				
Low	2190(79.3)	1753(79.3)	437(79.0)	0.923
High	573(20.7)	457(20.7)	116(21.0)	
Alkaline phosphate				
Low	1388(50.2)	1117(50.6)	271(49.0)	0.549
High	1375(49.8)	1093(49.4)	282(51.0)	
Bilirubin				
Low	2705(97.9)	2163(97.9)	542(98.0)	0.971
High	58(2.1)	47(2.1)	11(2.0)	
PNI				
Low	923(33.4)	737(33.3)	186(34.6)	0.938
High	1840(66.6)	1473(66.7)	367(66.4)	
SII				
Low	2987(75.5)	1678(75.9)	409(74.0)	0.364
High	676(24.5)	532(24.1)	144(26.0)	
SIRI				
Low	1948(70.5)	1558(70.5)	390(70.5)	1.000
High	815(29.5)	652(29.5)	163(29.5)	
MLR				
Low	1873(67.8)	1501(67.9)	372(67.3)	0.809
High	890(32.2)	709(32.1)	181(32.7)	
PLR				
Low	1980(71.7)	1584(71.7)	396(71.6)	1.000
High	783(28.3)	626(28.3)	157(28.4)	
NLR				
Low	1896(68.6)	1520(68.8)	376(68.0)	0.760
High	867(31.4)	690(31.2)	177(32.0)	
IPI risk group				
Low	702(25.4)	566(25.6)	136(24.6)	0.650
Low intermediate	775(28.0)	608(27.5)	167(30.2)	
High intermediate	729(26.4)	589(26.7)	140(25.3)	
High	557(20.2)	447(20.2)	110(19.9)	
Revised IPI risk group				
Low	174(6.3)	144(6.5)	30(5.4)	0.403
Intermediate	1303(47.1)	1030(46.6)	273(49.4)	
High	1286(46.5)	1036(46.9)	250(45.2)	
NCCN-IPI risk group				
Low	197(7.1)	163(7.4)	34(6.1)	0.783
Low intermediate	1111(40.2)	885(40.0)	226(40.9)	
High intermediate	1159(42.0)	927(42.0)	232(42.0)	
High	296(10.7)	235(10.6)	61(11.0)	

Note: MLR, monocyte-to-lymphocyte ratio; NLR, Neutrophil-to-Lymphocyte Ratio; PLR, Platelet-to-Lymphocyte Ratio; PNI, Prognostic nutrition index; SII, Systemic Immune-Inflammation Index; SIRI, Systemic Inflammation Response Index, IPI; International Prognostic Index, R-IPI; Revised-IPI, NCCN-IPI; National Comprehensive Cancer Network-IPI

Supplementary Table S5: Prognostic factors retained and rejected by final Boruta feature selection methods

	meanImp	medianImp	minImp	maxImp	normHits	decision
Age	-0.92363377	-1.27850679	-2.1182816	0.7949624	0.00000000	Rejected
Sex	-0.05801432	-0.07548467	-1.9219759	1.4012969	0.00000000	Rejected
Performance	10.33358740	10.40072201	3.7647616	13.6147257	1.00000000	Confirmed
Subtype	0.18284638	0.29982939	-2.3505916	1.7471350	0.00000000	Rejected
LDH	7.93526467	8.06870400	4.0231958	11.6550665	0.98989899	Confirmed
ALP	2.13940974	2.06956618	-0.5643595	5.0873281	0.27272727	Rejected
Bulk	3.63488970	3.62889352	0.7872201	7.2765812	0.75757576	Confirmed
Creatinine	1.37925248	1.44894035	-0.1006086	2.2401776	0.02020202	Rejected
Calcium	0.80964862	0.55745898	-1.1650404	2.4210014	0.02020202	Rejected
Bilirubin	1.69367671	1.62636944	-1.0063741	4.0966156	0.09090909	Rejected
Albumin	8.09928709	7.96702409	5.1647749	11.0384770	1.00000000	Confirmed
white_cell_count	6.42310265	6.45154322	3.5877657	9.1489142	1.00000000	Confirmed
Stage	11.89631865	11.90470236	8.6354643	15.8720874	1.00000000	Confirmed
Anemia	6.58710525	6.61466098	3.3104262	9.3836398	0.98989899	Confirmed
B_symptoms	3.00188511	3.19462434	-0.7152574	6.0729337	0.59595960	Confirmed
Extranodalnumbers	0.94429994	0.98770328	-0.9131637	2.7267707	0.01010101	Rejected
SII	4.95802282	4.84526636	1.2786050	9.1235251	0.94949495	Confirmed
SIRI	5.43262252	5.39252745	2.2631398	8.5850675	0.98989899	Confirmed
BMI	-0.32556875	-0.42676262	-2.2512834	1.8237404	0.00000000	Rejected
PLR	3.82580259	3.73215535	0.3688318	6.8339763	0.80808081	Confirmed
PNI	10.08155840	10.10346581	6.9330029	12.8792759	1.00000000	Confirmed
MLR	6.79971621	6.93212466	3.4425284	9.9014133	1.00000000	Confirmed
NLR	4.47493888	4.49973527	1.5997666	6.9667670	0.92929293	Confirmed
BCL2	3.00410643	2.94114246	-1.1736889	6.9351417	0.59595960	Confirmed
BCL6	1.87805019	1.92294536	-0.5407642	4.7173101	0.27272727	Rejected

Note: MLR, monocyte-to-lymphocyte ratio; NLR, Neutrophil-to-Lymphocyte Ratio; PLR, Platelet-to-Lymphocyte Ratio; PNI, Prognostic nutrition index; SII, Systemic Immune-Inflammation Index; SIRI, Systemic Inflammation Response Index,

Supplementary Table S6: Multivariable logistic regression in training data dataset

Variables	AOR (95% CI)
ECOG performance status	
0 or 1	1
2-4	1.69(1.29, 2.21)**
Stage	
I/II	1
III/IV	2.07(1.59, 2.72)**
LDH	
Normal	1
Elevated	1.46(1.17, 1.82)**
Albumin	
High	1
Low	1.13(0.89, 1.44)
BCL2 expression	
Negative	1
Positive	1.30(1.05, 1.62)*
B-symptoms	
Absent	1
Present	0.97(0.76, 1.24)
Bulk disease	

No	1
Yes	1.21(0.98, 1.50)
Anemia	
No	1
Yes	1.29(1.03, 1.63)*
Prognostic nutrition index	
Low	1.22(0.90, 1.67)
High	1
Systemic Immune-Inflammation Index	
Low	1
High	1.57(1.08, 2.28)*
Systemic Inflammation Response Index	
Low	1
High	1.27(0.88, 1.83)
Monocyte to lymphocyte ratio	
Low	1
High	1.12(0.81, 1.56)
Platelet to Lymphocyte Ratio	
Low	1
High	1.19(0.85, 1.66)
Neutrophil to Lymphocyte Ratio	
Low	1
High	1.03(0.71, 1.49)
White blood cell count	1.001(0.997, 1.004)

Note: * p value <0.05, ** p value <0.001, AOR; Adjusted Odds Ratio.

Supplementary Table S7: Performance of machine learning algorithms according to different data imbalance management methods

Algorithms	Unbalanced	Undersampling	Oversampling	ROSE	SOMTE
LR	0.70	0.69	0.70	0.68	0.69
RF	0.61	0.68	0.69	0.68	0.69
KNN	0.68	0.68	0.69	0.64	0.68
SVM	0.49	0.68	0.69	0.68	0.69
NB	0.70	0.69	0.70	0.69	0.70
GBM	0.69	0.68	0.69	0.68	0.68
XgBoost	0.69	0.68	0.70	0.66	0.69
IPI	0.65	0.65	0.65	0.60	0.65
R_IPI	0.61	0.61	0.61	0.61	0.61
NCCN_IPI	0.63	0.63	0.63	0.63	0.63

Note: ROSE, Oversampling of Examples Technique; SMOTE, Synthetic Minority Oversampling Technique, IPI; International Prognostic Index, R-IPI; Revised-IPI, NCCN-IPI; National Comprehensive Cancer Network-IPI, LR; logistic regression, RF; random forest, XgBoost; extreme gradient boosting, KNN; K-nearest neighbour, GBM; gradient boosting, SVM; support vector machine, NB; Naïve Bayes

LDH; Lactate Dehydrogenase, ECOG PS; Eastern Cooperative Oncology Group performance status, IPI; International Prognostic Index, R-IPI; Revised-IPI, NCCN-IPI; National Comprehensive Cancer Network-IPI, SII; Systemic Immune-Inflammation Index

Supplementary Figures

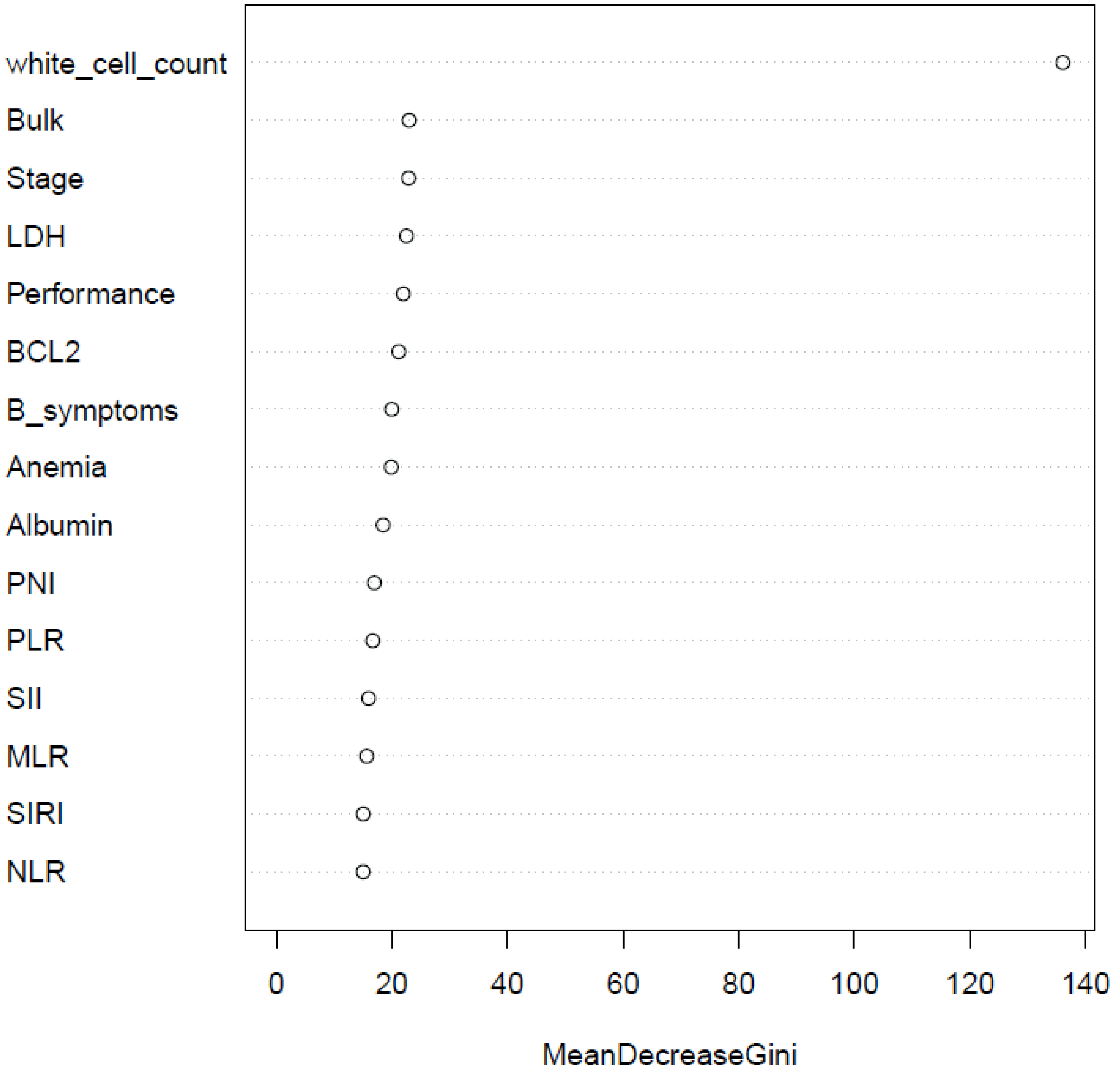

Supplementary Figure S1: Features ranking based on random forest algorithm. LDH, lactate dehydrogenase; MLR, monocyte-to-lymphocyte ratio; NLR, Neutrophil-to-Lymphocyte Ratio; PLR, Platelet-to-Lymphocyte Ratio; PNI, Prognostic nutrition; SII, Systemic Immune-Inflammation Index; SIRI, Systemic Inflammation Response Index

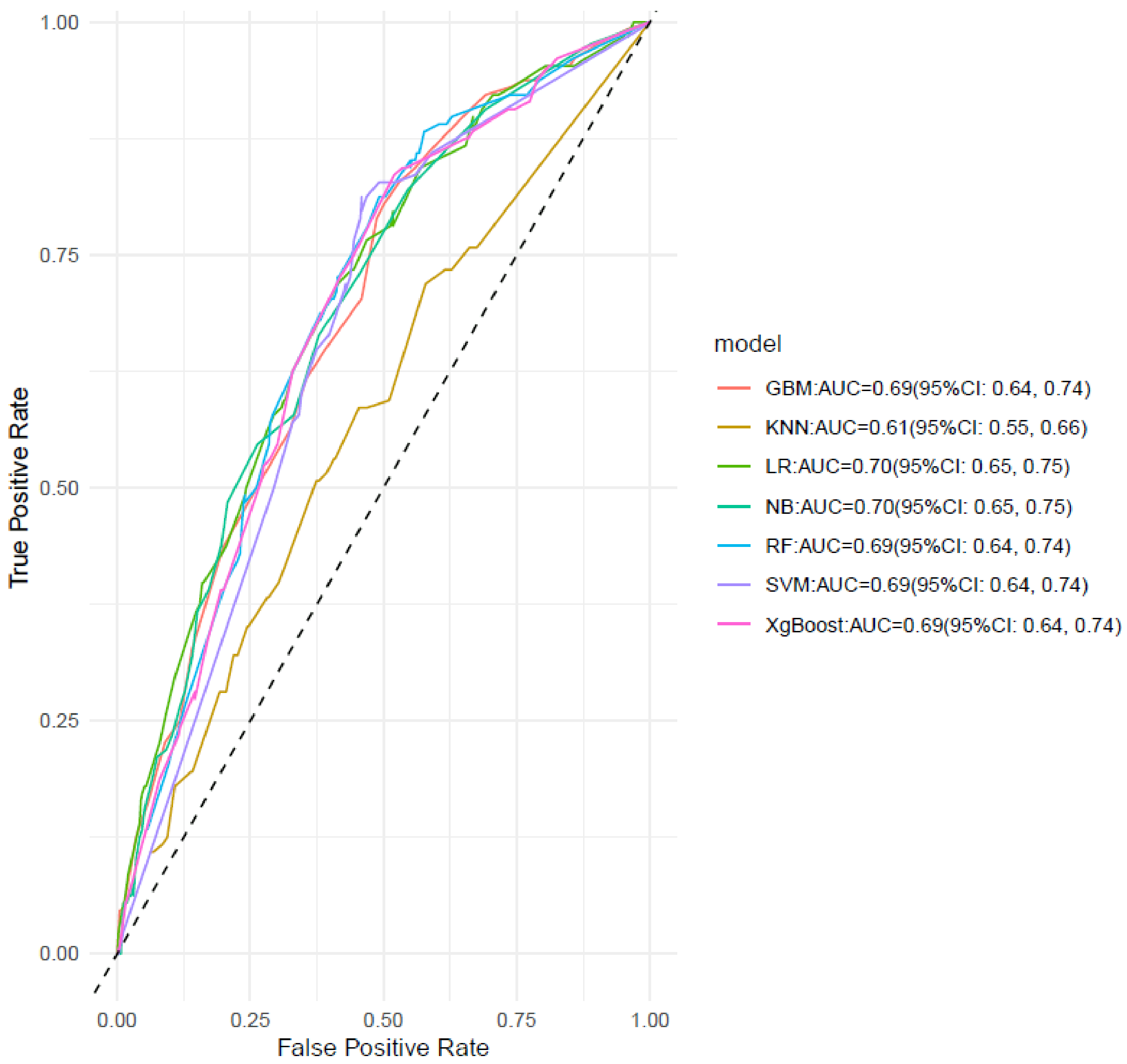

Supplementary Figure S2: Area under the curve (AUC) for machine learning algorithms with features selected using combined methods in the testing data set, balanced with SMOTE. LR; logistic regression, RF; random forest, XgBoost; extreme gradient boosting, KNN; K-nearest neighbour, GBM; gradient boosting, SVM; support vector machine, NB; Naïve Bayes

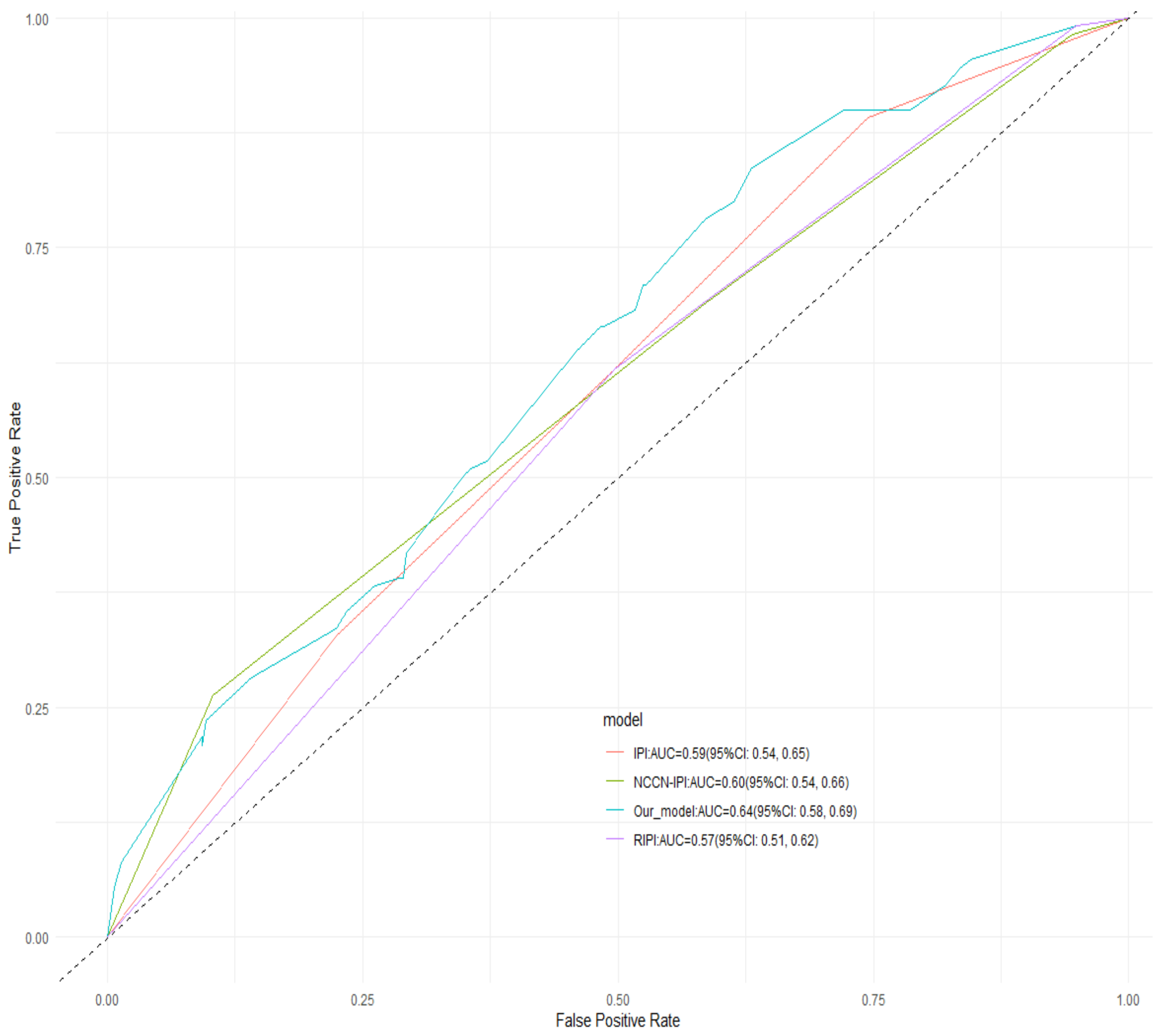

Supplementary Figure S3: Area under the curve (AUC) for our model (Nomogram) and existing tools in the testing data set for Diffuse large B-cell lymphoma. IPI; International Prognostic Index, R-IPI; Revised-IPI, NCCN-IPI; National Comprehensive Cancer Network-IPI.
